# Genomic, phenotypic, and clinical safety of *Limosilactobacillus reuteri* ATCC PTA 4659

**DOI:** 10.1093/jimb/kuad041

**Published:** 2023-11-16

**Authors:** Malin Sendelius, Jakob Axelsson, Peidi Liu, Stefan Roos

**Affiliations:** BioGaia AB, SE- 241 38 Eslöv, Sweden; BioGaia AB, SE- 241 38 Eslöv, Sweden; MetaboGen, SE- 411 26, Gothenburg, Sweden; BioGaia AB, SE- 103 64, Stockholm, Sweden; Department of Molecular Sciences, Uppsala BioCenter, Swedish University of Agricultural Sciences, Box 7015, SE- 750 07, Uppsala, Sweden

**Keywords:** Probiotics, *Limosilactobacillus reuteri*, *L. reuteri* ATCC PTA 4659, Safety assessment

## Abstract

Evaluating the safety of probiotic microorganisms is an important part of the development of probiotic products. In this study, we have performed a systematic safety assessment of *Limosilactobacillus reuteri* American Type Culture Collection (ATCC) PTA 4659 based on genome analysis, antibiotic susceptibility testing, phenotypic characterization, and a human clinical safety study. Genome sequence analysis showed that the strain is free from virulence and antibiotic resistance genes. Connected to this, phenotypic characterization showed that the strain is susceptible to the main classes of antibiotics. *Limosilactobacillus reuteri* ATCC PTA 4659 was shown to produce histamine, which has previously been described as an anti-inflammatory mediator produced by certain *L. reuteri* strains. However, the amount of histamine, a biogenic amine, poses no safety concern of a potential product. The strain was investigated in a human clinical safety study and was shown to survive passage through the gastrointestinal tract, both when administered at high [1 × 10^11^ colony-forming units (CFU)/day] and low doses (1 × 10^9^ CFU/day). The clinical safety evaluation showed that the doses administered are safe for human consumption. Furthermore, carbohydrate utilization, mucus adhesion, and tolerance to acid and bile were studied. It was shown that *L. reuteri* ATCC PTA 4659 has a very high adhesion to mucus and tolerance to both gastric pH and bile, all potentially important properties for a probiotic strain. Altogether, this study has demonstrated that *Limosilactobacillus reuteri* ATCC PTA 4659 is safe for human consumption and along with its phenotypic characteristics and previously described anti-inflammatory effects, makes it a promising strain for future probiotic development. NCT01033539

## Graphical Abstract

**Graphical Abstract:**
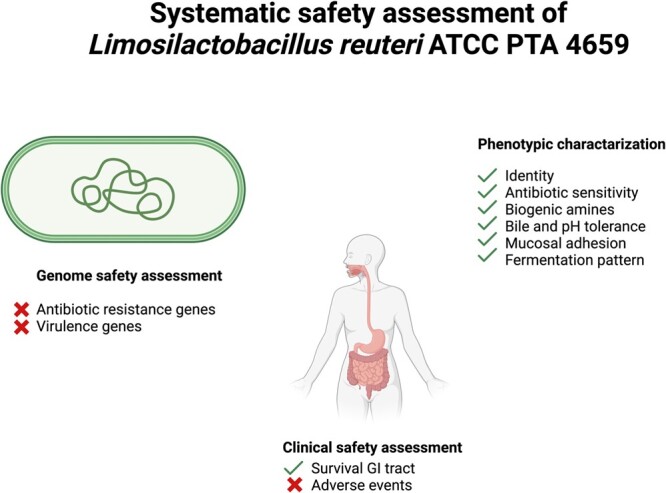
This is a systematic safety assessment of *Limosilactobacillus reuteri* ATCC PTA 4659 through genome analysis, phenotypic characterization, and a human clinical safety study.

## Introduction

One of the challenges developing probiotic products is how to select the bacterial strains. Studies of basic characteristics, evaluation in both *in vitro* and *in vivo* models, and comparisons with strains already tested in clinical studies can all be important parts of strain selection. Having a reliable identification method and performing a risk assessment are both mandatory, while the main part of the selection pipeline can be designed according to the intended use of the strain.

*Limosilactobacillus reuteri* is one of the most well-studied probiotic bacterial species described in a wide range of conditions, such as infantile colic and diarrhea, mainly using products containing strain DSM 17938 (Mu et al., 2018; Yu et al., 2023). Other well studied strains include American Type Culture Collection (ATCC) PTA 5289 (Lr5289), ATCC PTA 6475 (Lr6475), and ATCC PTA 4659 (Lr4659). Lr5289 has, together with DSM 17938, been described to ameliorate both oral cavity disorders and upper respiratory tract infections (Maya-Barrios et al., 2021; Schlagenhauf et al., 2020). Lr6475 has been shown to reduce bone loss (Nilsson et al., 2018) and, together with DSM 17938, to reduce antibiotic-associated side effects during treatment of *Helicobacter pylori* infections (Francavilla et al., 2014). In addition, *L. reuteri* strains have been found to have a solid safety profile (Mu et al., 2018; Yu et al., 2023) and the species consequently has a Qualified Presumption of Safety (QPS) status (Koutsoumanis et al., 2023).

In this study, we describe a basic characterization and safety assessment of Lr4659. This strain has previously been described in both preclinical and clinical studies, but the basic characterization and safety assessment have not been previously published. The strain belongs to the same phylogenetic clade of *L. reuteri* as Lr5289 and Lr6475 (Walter et al., 2011). This clade also includes the type strain *L. reuteri* DSM 20016 that has recently been classified as *L. reuteri* subsp. *reuteri* (Li et al., 2021). One of the most investigated attributes of probiotic strains is the ability to inhibit the growth of pathogenic microorganisms. It has been demonstrated that Lr4659, as well as Lr5289 and Lr6475, inhibit the growth of several bacterial pathogens by producing reuterin. Other lactobacilli were also shown to be inhibited, but to a significantly lesser degree (Spinler et al., 2008).

The effect of Lr4659 has been investigated in an obesity and fatty liver mouse model. Mice that were administered the strain gained significantly less body weight, adipose tissue, and liver weights, along with lower serum insulin levels than the control mice. The strain also increased the expression of carnitine palmitoyltransferase 1a in the liver, suggesting a mechanism of how gut bacteria can affect adiposity (Fåk & Bäckhed, 2012).

A signature feature of Lr5289, Lr6475, and Lr4659 is their anti-inflammatory effect, partly mediated by histamine (Ganesh et al., 2018; Haileselassie et al., 2013; Liu et al., 2010; Thomas et al., 2012; Versalovic et al., 2008). Interestingly, Lr4659 has been shown to cause a more pronounced amelioration of LPS-induced inflammation in the ileum of rat pups indicated by a strong downregulation of IL-13; (Liu et al., 2010) and a larger increase of Peyer's patches population of B cells than Lr6475. So even though the strains are very similar, they seem to interact with the immune system in slightly different ways.

The strong anti-inflammatory effects demonstrated in preclinical studies, combined with the safety status of *L. reuteri* and similarities to strains with proven clinical effects, were the reasons for evaluation of the immune-modulatory capabilities of Lr4659 in two separate randomized clinical studies (Ojetti et al., 2022; Petruzziello et al., 2019). The effects of the probiotic intervention targeted both the active inflammatory response and the downstream events. The strain proved to possess anti-inflammatory properties as evidenced by plasma C-reactive protein (CRP) in both studies and the response was studied in two different settings. In the first study, patients with a high degree of fever, were treated with antibiotics along with the administration of probiotics and in the second study, patients had a lower degree of fever and were not using antibiotics. In both studies, a total number of 207 patients diagnosed with acute uncomplicated diverticulitis were administered active [containing 5 × 10^8^ colony-forming units (CFU) Lr4659] or placebo capsules twice daily. Systemic inflammation, measured as plasma CRP 72 hr after hospital admission, was reduced in both studies. During antibiotic treatment, CRP was significantly lowered from an evenly distributed baseline for both groups of about 70 mg/L to a concentration of 23 mg/L for the probiotic group vs. 45 mg/L in the placebo group (Petruzziello et al., 2019). In the study when no antibiotics were used, the percent of change was largest in the probiotics group (59% vs. 40% of the placebo control) (Ojetti et al., 2022). Local inflammation, measured by fecal calprotectin 72 hr after hospital admission, was investigated in patients not treated with antibiotics and was found to be significantly lowered, as evidenced by a reduction of 17% in the probiotic group compared to 11% of the placebo group (Ojetti et al., 2022). Pain, because of intestinal inflammation, was also significantly lower in the probiotic group compared to the placebo group 3–10 days after hospital admission when measured in patients receiving antibiotics (Petruzziello et al., 2019). No adverse events were reported in either of the two studies.

As mentioned above, the present study describes the basic characterization and safety assessment of Lr4659. This includes analyses of basic phenotypic characteristics, such as stress tolerance, adhesion to mucus, and metabolic capacity, as well as safety assessment by genomic safety and clinical safety evaluation.

## Materials and Methods

### Bacterial Strains and Culture Conditions

The strain Lr4659, originally designated MM2-3, was isolated from the breast milk of a mother in Tampere, Finland (Karvonen et al., 1998). The strain was first identified as *Lactobacillus reuteri* (Walter et al., 2011). A recent revision of the taxonomy has changed the identity to *Limosilactobacillus reuteri* subsp. *reuteri* (Li et al., 2021). Upon being deposited to the ATCC, the strain was designated ATCC PTA 4659.

Other strains used in this study are the *L. reuteri* strains ATCC PTA 5289 (Lr5289; previously designated FJ1), ATCC PTA 6475 (Lr6475; previously designated MM4-1a), and DSM 17938. They are used in commercial probiotic products and were obtained from BioGaia AB (Stockholm, Sweden). *L. reuteri* ATCC 55730 (previously designated SD2112) was obtained from BioGaia AB (Stockholm, Sweden).

All strains were grown in DeMan, Rogosa, and Sharpe (MRS) broth or MRS agar (Oxoid or Merck) at 37°C.

### Genomic Analysis

#### Whole-Genome Sequencing and Plasmid DNA Extraction

DNA of Lr4659 was isolated and sequenced with both Illumina MiSeq (short reads) and PacBio (long reads). The total genome coverage was 10x and the reads were assembled using Quiver v2.3. The genome sequence was deposited to GenBank (http://www.ncbi.nlm.nih.gov/genbank/) and has the accession no. GCA_030418275.1.

The average nucleotide identity (ANI) value was calculated based on BLAST using the JSpeciesWS ANI calculator (Richter et al., 2016).

Plasmid DNA was extracted from bacteria grown overnight using QIAprep Spin Miniprep Kit (Qiagen) following the manufacturer´s instructions except in the first step where 20 mg/mL lysozyme (Sigma–Aldrich) and 100 U/mL mutanolysin (Sigma–Aldrich) were added to the buffer. Extracted plasmids were separated by agarose gel electrophoresis.

#### Genome Functional Annotation and Comparison

To make the functional annotation comparable across different strains, the coding sequences (CDS) from each strain were annotated using the eggNOG (Huerta-Cepas et al., 2016) tool (v5) standalone pipeline (https://github.com/eggnogdb/eggnog-mapper) with mapping mode ‘HMMER’ and ‘Bacteria’ taxonomic level with other parameters untouched. The *L. reuteri* strains used were Lr4659, Lr6475 (NCBI accession number: GCA_000159475.2 (MM4-1a)), JCM 1112 (type strain; same strain as DSM 20016; NCBI accession number: GCF_000010005.1), and SD2112 (same strain as ATCC 55730; NCBI accession number: GCF_000159455.2). The Clusters of Orthologous Genes (COGs) discovered by eggNOG were compared across the strains, with special attention to the COG ‘V’ category (Defense mechanism).

#### Identification of Antibiotic Resistance Genes, Virulence Factors, and Genomic Islands

The Lr4659 genome was screened for antibiotic resistance factors using the Resistance Gene Identifier (RGI), which is part of the Comprehensive Antibiotic Resistance Database (CARD) (Alcock et al., 2020; McArthur et al., 2013). CARD is a platform containing a reference database on the molecular basis of antimicrobial resistance genes. The Lr4659 genome was submitted to the RGI CARD webserver (accessed April 2021) using the ‘Strict’ setting. As a complement to CARD, the ResFinder database (http://genepi.food.dtu.dk/resfinder) using ResFinder 4.1 software (Bortolaia et al., 2020) (accessed April 2021) was also used to identify putative antimicrobial resistance genes.

To identify genomic islands and virulence factors, IslandViewer4 (http://www.pathogenomics.sfu.ca/islandviewer/, accessed April 2021) was used. IslandViewer4 (Bertelli et al., 2017) uses a set of tools and databases to identify virulence factors, such as the Virulence Factor Database (VFDB) (Chen et al., 2012), the bacterial bioinformatics database and analysis resource PATRIC (Wattam et al., 2014) and Victor's virulence factors (Victors: a web-based knowledge base of virulence factors in human and animal pathogens) (Sayers et al., 2019).

### Safety Assessment

#### Determination of Minimum Inhibitory Concentrations (MICs)

Lr4659 was assessed for susceptibility to ampicillin, chloramphenicol, clindamycin, erythromycin, gentamicin, kanamycin, streptomycin, and tetracycline according to guidelines from the European Food Safety Authority (EFSA) (Rychen, et al., 2018). The tests were performed at the Swedish National Veterinary Institute (Uppsala, Sweden). VetMIC Lact-1 and VetMIC Lact-2 microdilution panels (the Swedish National Veterinary Institute) were used in an anaerobic atmosphere following ISO 10932:2010 (ISO, 2010). Bacterial cells were diluted to a density of 5 × 10^5^ CFU/mL in LSM broth (Klare et al., 2005). The panels were inoculated with 100 μL/well of bacterial suspension and incubated at 37°C for 48 hr. *Lacticaseibacillus paracasei* ATCC 334 was tested in tandem with Lr4659 to verify the methodology performed in this study, and it exhibited MICs within accepted ranges in ISO 10932:2010 (ISO, 2010). MIC values were compared with cut-off values for *L. reuteri* strains defined by EFSA (Rychen, et al., 2018).

#### Biogenic Amines

Production of the biogenic amines, histamine, tyramine, cadaverine, and putrescine was investigated. The HPLC analyses were performed at Eurofins Food & Feed Testing Sweden (Lidköping, Sweden). Industrially produced lyophilized Lr4659 culture powder obtained from BioGaia AB (Stockholm, Sweden) was used to analyze the production of biogenic amines according to Smělá et al. (2003).

#### Clinical Safety and Tolerability Assessment

The study was designed as a randomized, double-blind, placebo-controlled clinical study performed by Good Food Practice, Uppsala, Sweden. The study protocol was reviewed and accepted by the ethical committee in Uppsala, Sweden (Dnr 2009/323). The study design was made publicly available and was registered at clinicaltrials.gov (NCT01033539).

In total, 30 participants were randomized into three groups, 6 participants in the placebo group, 12 participants in the low dose group, and 12 participants in the high dose group. All study participants, adult, free-living healthy volunteers of both genders, received either active or placebo product for 28 days, followed by a follow-up period of 14 days. In total, 29 subjects completed the study and one subject in the low dose group withdrew during the study.

Inclusion criteria were; males and females, age 18–65 years, BMI 19–33, Hb ≥ 120 g/L for women, and ≥ 130 g/L for men, healthy as assessed by the screening laboratory tests, signed informed consent, and biobank consent. Exclusion criteria were previous participation in a clinical study 90 days prior to screening visit, use of oral antibiotics and probiotics during 2 weeks prior to baseline visit, intake of other probiotics during the study period, pregnancy, and lack of suitability for participation for any reason as judged by the study personnel.

The two active study products contained Lr4659 and maltodextrin in a sachet. Active study product Low dose contained a dose per sachet of Lr4659 1 × 10^9^ CFU (1.9 × 10^9^ CFU/sachet at study start and 1.5 × 10^9^ CFU/sachet at end of study). Study product High dose contained a dose per sachet of Lr4659 1 × 10^11^ CFU (1.6 × 10^11^ CFU/sachet at study start and 1.4 × 10^11^ CFU/sachet at end of study). The placebo product was composed of identical formulation, maltodextrin without Lr4659 culture powder.

The primary objective of the study was to determine the effects of the probiotics on the recovery of Lr4659 in fecal samples at baseline (day 0) and after 7, 14, and 28 days supplementation as well as after an additional 14 day (total 42 days) washout period, as compared to placebo. The primary endpoint was the change of Lr4659 in fecal samples from baseline to day 28 of treatment. Secondary objectives of the study included quantification of blood chemistry (P-ALAT, P-ASAT, P-gGT, P-Creatinine, S-TSH, total bilirubin, Evf, Hb, Lkc, MCH, MCHC, MCV, Trc, Erc, and HS-CRP), blood pressure, and heart rate after 7, 14, and 28 days supplementation as well as 14 days follow up, compared to placebo. Adverse events (AEs) were recorded on day 7, day 14, day 28, and day 42.

The fecal samples, still blinded, were analyzed for live Lr4659 by using the following method. Fecal samples were thawed at room temperature and 0.3 g was suspended in 2.7 mL phosphate buffered saline (PBS). This suspension was further diluted in PBS and plated on Rogosa agar (Oxoid). Plates were incubated anaerobically (AnaeroGen) at 45°C for 48 hr, selecting for thermotolerant lactobacilli as *L. reuteri*. The colonies were counted and 5–10 colonies (with the typical appearance large, white, and shiny) per sample were picked to two new Rogosa plates, and after anaerobic incubation at 37° for 48 hr, further analyzed by a reuterin overlay assay and a strain specific PCR assay. The reuterin overlay assay was performed according to Rosander et al., 2008. In short, one of the plates was overlaid with 500 mM glycerol agar (1% agar) and incubated at 37°C for 30 min. Reuterin was detected by the addition of 5 mL 2,4-dinitrophenylhydrazine (0.1% in 2 M HCl). After 3 min incubation, the solution was poured off and 5 mL 5 M KOH was added. Red zones around the colonies demonstrated the production of reuterin. For the PCR analysis, 1 μL of each isolate was collected with a sterile plastic loop and suspended in 100 μL sterile water. PCR were run by using PuReTaq Ready To Go PCR beads (GE HealthCare) and the primer pair SNP5f, GCTAAAGCCTTGCTGAAACG, and SNP5_4659, CGCATACGGGGTGTA, directed against SNP5 in the Lr4659 genome (Walter et al., 2011; 0.4 μM of each primer). Bacterial suspension (0.5 μL) was added to the mix and the PCR reaction was performed by running the program 95°C, 7 min; 35x (95°C, 30 s; 62°C, 30 s; 72°C, 30 s); 72°, 10 min. The PCR products (141 bp) were visualized by using standard agarose gel electrophoresis. Lr4659 was used as a positive and Lr5289 as a negative control in the PCR. Isolates positive both in the reuterin assay and the PCR were concluded to be Lr4659.

Blood chemistry parameters were analyzed according to routine at Uppsala University Hospital, Uppsala, Sweden.

### Physiological Characterization

#### Morphology

The bacterial morphology of Lr4659 was observed by light microscopy (ECLIPSE Ci, Nikon).

#### Carbohydrate Utilization

The carbohydrate utilization ability was determined using an API 50 CHL kit (BIOMÉRIEUX) according to the manufacturer's instruction. Bacterial cells grown on MRS agar (Oxoid) were suspended in API 50 CHL medium to a cell density corresponding to McFarland Standard No. 2 (BIOMÉRIEUX). The suspended cells were inoculated into the well-type plate provided by the manufacturer, and the plate was incubated at 37°C for 48 hr. The carbohydrate utilization ability was assessed by determining color changes.

#### Acid Tolerance

The survival at pH 2 was tested according to Wall et al. (2007) in synthetic gastric fluid described by Cotter et al. (2001) that was modified by not adding enzymes. Duplicate samples were taken after 50 and 90 min.

#### Bile Tolerance

Cells were incubated in a microtiter plate at 37°C for 27–30 hr in MRS medium (Merck) with and without the addition of 0.2% bovine bile (B3883, Sigma–Aldrich) using a short shaking step every 30 min. The optical density (OD) was measured at 620 nm using a Multiskan FC Microplate Photometer (Thermo Fisher Scientific). The growth in the presence of bile was compared to growth without bile.

#### Adhesion

The adhesion assay was an adaptation of a previously described method (Roos et al., 2000). In brief, mucus was scraped off from the mucosa of a porcine small intestine, followed by two centrifugations (11 000 x *g* for 10 min and 26 000 x *g* for 15 min). The crude mucus preparation was diluted to OD_280_ 0.1 in PBS (pH 6.0) and added to each well on the Nunc Maxisorb plate (Nalgene-Nunc, Thermo Fisher Scientific), which was then incubated at 4°C overnight under slow rotation. The wells containing mucus were washed three times with PBS (pH 6.0) + 0.05 % Tween 20 (PBST) and blocked for 60 min with PBS + 1 % Tween 20 (pH 6.0). The suspension was diluted to OD_600_ 0.5 followed by two additional washes with PBST upon which the bacteria were added to the wells (suspended in PBST). A reference at timepoint 0 was removed before addition of bacteria to the wells. The bacteria were incubated in the plate for 4 hr at 37°C with slow agitation. Non-adherent bacteria were removed by washing with PBST, and viable adhered bacteria were treated with trypsin ethylenediaminetetraacetic acid (EDTA, 0.25%) for 30 min to release them from the plate followed by serial dilutions and plating on MRS plates. The plates were anaerobically incubated at 37°C for 48 hr before viable counts were determined.

## Results and Discussion

### Whole Genome Sequence Analysis

The genome of Lr4659 was sequenced, resulting in two contigs constituting a total size of 2 096 828 bp, having a 38.9% GC content and 2111 CDS. No plasmids were detected in the genome, which was confirmed in a plasmid extraction analysis (Supplementary Figure S1). Analysis of the whole genome sequence confirmed the identity of the strain as *Limosilactobacillus reuteri* with an ANI value of 99.99% between Lr4659 and the type strain *L. reuteri* subsp. *reuteri* JCM 1112. Consequently, similar to the analysis by Li et al. (2021), Lr4659 was classified as *L. reuteri* subsp. *reuteri*.

### Genomic Analysis of the Safety-Related Genes

We first annotated the CDS in the genomes of Lr4659, Lr6475, JCM 1112, and SD2112, whereafter the COG functional categories were compared across the strains (Supplementary Table S1). The COG analysis revealed that the overall functional composition of Lr4659 is very similar to the strains used for comparison. The COG category ‘V’, i.e. the ‘Defense mechanism’ group may have a potential safety interest, such as antibiotic resistance. Thus, the COGs found in this specific group in all the genomes were therefore compared across the strains (Supplementary Table S2). The overall COG pattern across all the analyzed well-known strains was found to be very similar, thus, there is no safety concern for Lr4659 regarding genome functional composition.

Next, the whole genome sequence of Lr4659 was analyzed for antibiotic resistance genes, virulence factors, and genomic islands (GIs). No antibiotic resistance genes were detected and in total 12 GIs were found. No genes encoding virulence factors or antibiotic resistance were found inside or outside the genomic islands. In conclusion, the genomic analysis of Lr4659 raises no safety concerns.

### Determination of MICs and Biogenic Amines

The MIC of the antibiotics; ampicillin, chloramphenicol, clindamycin, erythromycin, gentamicin, kanamycin, streptomycin, and tetracycline were tested for Lr4659. The MIC values of all tested antibiotics were below the cut-off limits defined by EFSA (Table 1).

**Table 1.** Minimum Inhibitory Concentrations of Antibiotics for Lr4659

Antibiotics	MIC (mg/L)	EFSA MIC (mg/L) Resistance Threshold*
Ampicillin	1	2
Chloramphenicol	4	4
Clindamycin	<0.03	4
Erythromycin	0.25	1
Gentamicin	<0.5	8
Kanamycin	16	64
Streptomycin	8	64
Tetracycline	16	32

* European Food Safety Authority (Rychen, et al., 2018)

Another potential health concern is production of the biogenic amines; histamine, tyramine, cadaverine, and putrescine (Ruiz-Capillas & Herrero, 2019). It has previously been shown that Lr4659 has a complete histidine decarboxylase gene cluster and that it is capable of producing histamine from histidine, like several other strains belonging to the same phylogenetic clade (Spinler et al., 2014). The production of histamine has in several studies been linked to the anti-inflammatory properties of Lr6475 (Ganesh et al., 2018; Hemarajata et al., 2013; Thomas et al., 2012) and most likely this is also the case for Lr4659. To ensure that the production of biogenic amines is not too high, the concentrations of histamine, tyramine, cadaverine, and putrescine were analyzed in lyophilized culture powder of Lr4659. The strain did not produce tyramine nor cadaverine, whereas histamine (222 mg/kg) and putrescine (4 mg/kg) were detected. The highest daily dose evaluated in the clinical safety study (in Materials and Methods) was 1 × 10^11^ CFU/day, which corresponds to around 1 g culture powder/day (data not shown). Thus, a daily dose of 1 × 10^11^ CFU Lr4659 would provide an intake of around 200 μg/day histamine and 4 μg/day putrescine. Acceptable levels of histamine have been reported to be below 50 mg/kg in fishery products (Ruiz-Capillas & Herrero, 2019) and 50–400 mg/kg in cheese (Benkerroum, 2016), which corresponds to an intake of approximately 2.5–20 mg histamine after eating a typical portion of fish (150 g) or cheese (50 g). The highest daily intake of histamine from Lr4659 (200 μg) is consequently below the acceptable intake of histamine from common food products (2.5–20 mg/day). Maximum tolerable levels of putrescine in cheese have been proposed to be at least 180 mg/kg (Benkerroum, 2016). An estimated daily intake of 50 g cheese corresponds thus to 9 mg putrescine, which is considerably higher than the high dose of Lr4659 (4 μg/day).

### Clinical Safety and Tolerability Assessment

Demographics and baseline characterization are summarized in Table 2. None of the baseline variables differed significantly between the study groups.

**Table 2.** Demographics and Disposition of Study Subjects

	**High**	**Low**	**Placebo**	**Total**
	**(*N* = 12)**	**(*N* = 12)**	**(*N* = 6)**	**(*N* = 30)**
Gender *n* (%)				
Female	11 (92)	8 (67)	4 (67)	23 (77)
Male	1 (8)	4 (33)	2 (33)	13
Age				
Mean (SD)	44.7 (15.0)	39.8 (16.2)	40.4 (18.3)	41.8 (15.8)
Median	49	32	36	44
Range	21–63	22–63	22–63	21–63
*N*	12	12	6	30
BMI				
Mean (SD)	24.0 (2.4)	23.4 (3.5)	22.5 (2.9)	23.5 (2.9)
Median	23.9	22.4	22	22.8
Range	19.4–27.6	19.4–29.4	20–27.9	19.4–29.4
*n*	12	12	6	30
Weight				
Mean (SD)	44.7 (15.0)	39.8 (16.2)	40.4 (18.3)	41.8 (15.8)
Median	65	68.5	61	65.5
Range	57–87	53–95	52–85	52–95
*n*	12	12	6	30
Height				
Mean (SD)	167 (7)	172 (5)	170 (8)	170 (7)
Median	164	171	169	168
Range	159–180	165–180	161–182	159–182
*n*	12	12	6	30

**Fig. 1.**
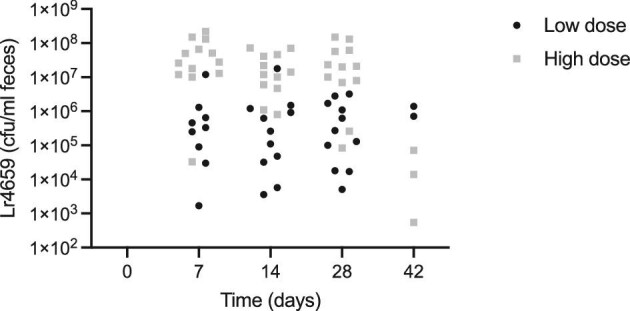
Recovery of Lr4659 as measured by CFU/ml feces.

Recovery of Lr4659 in feces was measured at baseline, day 7, day 14, day 28, and at follow-up on day 42 (Fig. 1). At baseline, none of the participants showed any detectable Lr4659 in feces and neither did any participants receiving placebo product throughout the study period. All participants had detectable levels of Lr4659 in feces on days 7, 14, and 28, except for two individuals who had no detectable levels on day 7. In two individuals, Lr4659 was recovered from feces after the 14-day washout period at day 42. In the individuals receiving the high dose, all showed Lr4659 in feces on day 7, 17, and 28, and three individuals had detectable levels after washout at day 42. These results clearly show that Lr4659 survives the gastrointestinal tract in humans but indicates that the strain only transiently colonizes the GI tract.

The safety assessment of blood chemistry included ALAT, ASAT, γGT, creatinine, THS, total bilirubin, hematology (Evf, Hb, Lkc, MCH, MCV, Trc, Erc), and marker for inflammation, (HS-CRP). Erc, HCMC, and thrombocytes were increased in the placebo group, but all were within the reference values. The Hb decreased by 8 g/L in the high dose group and by 4 g/L low dose and placebo groups between day 0 and day 28. All values were within the reference range and did not differ significantly between any of the groups. One subject in the placebo group displayed elevated CRP, which was ruled due to pneumonia. All other variables did not differ between the groups and were within reference values.

Systolic and diastolic blood pressure as well as heart rate were recorded at each visit. No significant differences between the groups could be detected (data not shown).

The occurrences of AEs are summarized in Table 3. A total of 10 subjects reported AEs and 4 subjects reported AEs related to the study product. One subject withdrew from the study due to an AE (flatulence). The types of AEs are described in Table 4. A total of 5 subjects reported infections during the study of which 4 were in the low dose group (3 common cold and 1 conjunctivitis) and 1 in the placebo group (pneumonia). None of the infections were deemed to be related to the study product. Five participants reported gastrointestinal disorders, three in the high dose and two in the low dose groups. One participant in the placebo group reported an AE due to allergy.

**Table 3.** Summary of Adverse Events

	**High**	**Low**	**Placebo**	**Total**
	(*N* = 12)	(*N* = 12)	(*N* = 6)	(*N* = 30)
Subjects with AE	2 (17)	6 (50)	2 (33)	10 (33)
Total number of AEs	3	8	2	13
Subjects with related AE	1 (8)	3 (25)	0	4 (13)
Total number of related AEs	2	5	0	7
Subjects discontinued due to AE	0	1 (8)	0	1 (3)

**Table 4.** Summary of Adverse Events by Type

	**High**	**Low**	**Placebo**	**Total**
	**(*N* = 12)**	**(*N* = 12)**	**(*N* = 6)**	**(*N* = 30)**
Infections	0	4 (33)	1 (17)	5 (17)
Gastrointestinal disorders	3 (25)	2 (17)	0	5 (17)
Asthma and allergy	0	0	1 (17)	1 (3)
Other	0	1 (8)	0	1 (3)

Subjects are only counted once per treatment for each row.

In conclusion, the results from the clinical study showed that Lr4659 survives passage through the gastrointestinal tract in humans, both when administered at a high (1 × 10^11^ CFU) and low dose (1 × 10^9^ CFU). The clinical safety evaluation of Lr4659 indicated that the doses administered are safe for human consumption.

### Physical and Physiological Characterization

Light microscopy images revealed that Lr4659 is a rod-shaped bacterium with 1–3 μm long cells, either single or in pairs (Supplementary Figure S2). The carbohydrate utilization pattern was analyzed (Supplementary Table S3) and found to be typical for *L. reuteri* subsp. *reuteri* strains (Li et al., 2021).

**Fig. 2.**
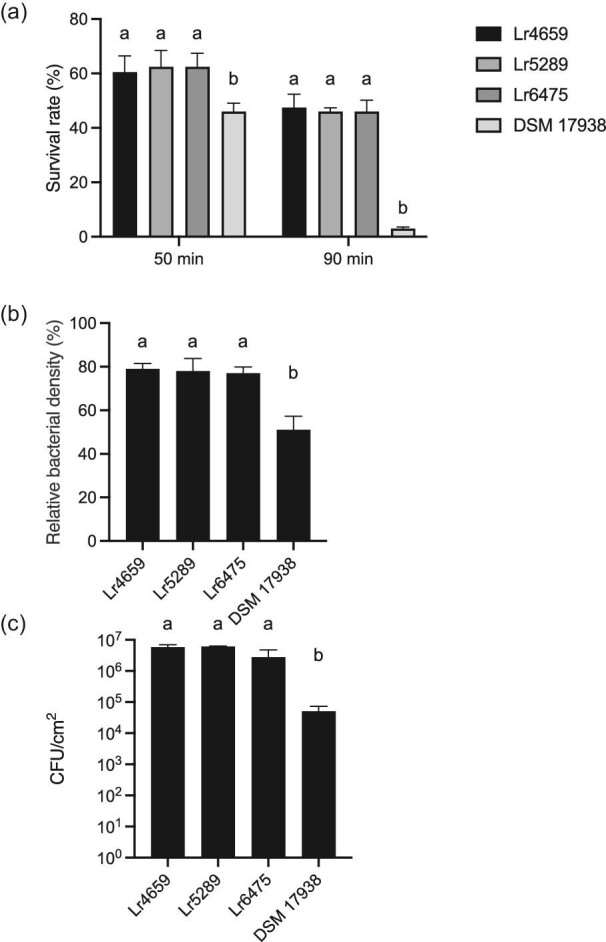
Tolerance characteristics and mucus binding of the *Limosilactobacillus reuteri* strains Lr4659, Lr5289, Lr6475, and DSM 17938. Columns with different letters are significantly different (*p* < 0.05). Statistical analysis by one-way analysis of variance (ANOVA), *p* < 0.05. (a) Acid tolerance was assessed by exposing the bacteria to synthetic gastric juice at pH 2.0 for 50 and 90 min. The columns show the percentage of bacteria surviving at pH 2.0 (mean of quadruplicates ± standard deviation). (b) Bile tolerance was determined by growth of *L. reuteri* strains in the presence of 0.2% bovine bile. The bile tolerance is expressed as the relative growth in DeMan, Rogosa and Sharpe (MRS) medium with bile compared to MRS without bile. The presented data is the mean of 2–3 replicates ± standard deviation. (c) Adhesion to mucus prepared from porcine small intestine. After allowing the strains to bind to mucus-coated surfaces, the number of bacteria were determined by trypsin release and plating. The columns show the number of CFU/cm^2^ of adherent bacteria (mean of triplicates ± standard deviation).

Orally ingested probiotic strains should preferably survive the passage of the stomach with its low pH and become active in the upper gastrointestinal tract, where there is a high concentration of bile with antimicrobial properties. Therefore, acid and bile tolerance of Lr4659 was investigated and compared with other probiotic strains of *L. reuteri*. Lr 4659 and two other *L. reuteri* subsp. *reuteri* strains survived well in synthetic stomach juice with pH 2.0, whereas *L. reuteri* DSM 17938 had a lower survival rate (Fig. 2a). However, it should be noted that this type of assay often is performed at higher pH (Ko et al., 2022) and that all strains had a good tolerance to low pH. Bile tolerance was analyzed by cultivation in MRS medium with and without addition of 0.2% bovine bile. All tested strains grew well in the presence of bile and Lr4659 and the other *L. reuteri* subsp. *reuteri* strains showed only 20% reduction, whereas DSM 17938 displayed around 50% reduction compared to growth without bile (Fig. 2b).

Another property of presumed importance for a probiotic strain is adhesion to the mucosa, which potentially can increase the intestinal residence time and interactions with host epithelial and immune cells. The ability to adhere to mucus prepared from porcine small intestine was therefore analyzed and Lr4659 as well as the other *L. reuteri* subsp. *reuteri* strains showed a high adhesion to mucus (Fig. 2c) with an almost complete coverage of the mucus coated surface (data not shown). Also in this analysis, these strains gave higher values than DSM 17938 (Fig. 2c). *Limosilactobacillus reuteri* strains Lr5289, Lr6475, and DSM 17938 have been evaluated in several clinical studies, many demonstrating a good probiotic effect (Mu et al., 2018; Nilsson et al., 2018; Schlagenhauf et al., 2016; Yu et al., 2023). The data presented show that Lr4659 is both stress tolerant and possesses an adhesive phenotype that is at least equal to these well-known probiotic strains.

## Conclusions

The results from this systematic safety evaluation, based on genome analysis, antibiotic susceptibility testing, phenotypic characterization, and a human clinical safety evaluation, demonstrate that Lr4659 is safe for human consumption. This along with previously described preclinical and clinical effects, provides strong evidence for the use of this strain in future probiotic products.

## Supplementary Material

kuad041_Supplemental_File
