# Supplementary material for: Genomic, phenotypic, and clinical safety of Limosilactobacillus reuteri ATCC PTA 4659
Source: J Ind Microbiol Biotechnol. 2023 Nov 16;50(1):kuad041. doi: 10.1093/jimb/kuad041 (PMC10689046; doi:10.1093/jimb/kuad041)
Supplement: kuad041_Supplemental_File [file kuad041_supplemental_file.zip › Supplementary figure legends.docx]

**Supplementary figure legends**

Supplementary Figure 1. Agarose gel electrophoresis showing extracted plasmids of L. reuteri strains 1) Lr6475; 2) Lr4659; 3) Lr5289; 4) DSM 17938; and 5) ATCC 55730. Lane 6 contains chromosomal DNA from L. reuteri ATCC 55730 and lane 7 the marker, phage lambda EcoRI/HindIII.

Supplementary Figure 2. Representative light microscope image of Lr4659. The size bar corresponds to 10 μm.
