# Supplementary material for: Genomic, phenotypic, and clinical safety of Limosilactobacillus reuteri ATCC PTA 4659
Source: J Ind Microbiol Biotechnol. 2023 Nov 16;50(1):kuad041. doi: 10.1093/jimb/kuad041 (PMC10689046; doi:10.1093/jimb/kuad041)
Supplement: kuad041_Supplemental_File [file kuad041_supplemental_file.zip › Supplementary tables.docx]

**Supplementary tables**

Supplementary Table 1. Comparison of COG functional categories of *L. reuteri* strain genomes. The COG categories were taken from the eggNOG database. Analyzed *L. reuteri* strains: Lr4659, Lr6475, JCM 1112 and SD2112.

| **Strain** | **Lr4659^1^** | | **Lr6475^2^** | | **JCM 1112^3^** | | **SD2112^4^** | |
| --- | --- | --- | --- | --- | --- | --- | --- | --- |
| **CDS** | 2111 | | 2095 | | 1986 | | 2213 | |
| **Category - Description** | #  gene | % | #  gene | % | #  gene | % | #  gene | % |
| J – Translation, ribosomal structure and biogenesis | 166 | 7.86 | 149 | 7.11 | 154 | 7.75 | 150 | 6.78 |
| A – RNA processing and modification | 0 | 0.00 | 0 | 0.00 | 0 | 0.00 | 0 | 0.00 |
| K – Transcription | 112 | 5.31 | 108 | 5.16 | 98 | 4.93 | 103 | 4.65 |
| L – Replication, recombination and repair | 202 | 9.57 | 198 | 9.45 | 131 | 6.60 | 250 | 11.30 |
| B – Chromatin structure and dynamics | 0 | 0.00 | 0 | 0.00 | 0 | 0.00 | 0 | 0.00 |
| D – Cell cycle control, cell division, chromosome partitioning | 32 | 1.52 | 31 | 1.48 | 31 | 1.56 | 28 | 1.27 |
| Y – Nuclear structure | 0 | 0.00 | 0 | 0.00 | 0 | 0.00 | 0 | 0.00 |
| V – Defense mechanisms | 19 | 0.90 | 20 | 0.95 | 20 | 1.01 | 21 | 0.95 |
| T – Signal transduction mechanisms | 36 | 1.71 | 36 | 1.72 | 34 | 1.71 | 33 | 1.49 |
| M – Cell wall/membrane/envelope biogenesis | 81 | 3.84 | 75 | 3.58 | 78 | 3.93 | 79 | 3.57 |
| N – Cell motility | 9 | 0.43 | 9 | 0.43 | 9 | 0.45 | 9 | 0.41 |
| Z – Cytoskeleton | 0 | 0.00 | 0 | 0.00 | 0 | 0.00 | 0 | 0.00 |
| W – Extracellular structures | 0 | 0.00 | 0 | 0.00 | 0 | 0.00 | 0 | 0.00 |
| U – Intracellular trafficking, secretion, and vesicular transport | 39 | 1.85 | 37 | 1.77 | 35 | 1.76 | 36 | 1.63 |
| O – Posttranslational modification, protein turnover, chaperones | 38 | 1.80 | 37 | 1.77 | 36 | 1.81 | 38 | 1.72 |
| X – Mobilome: prophages, transposons | 0 | 0.00 | 0 | 0.00 | 0 | 0.00 | 0 | 0.00 |
| C – Energy production and conversion | 80 | 3.79 | 75 | 3.58 | 76 | 3.83 | 72 | 3.25 |
| G – Carbohydrate transport and metabolism | 98 | 4.64 | 95 | 4.53 | 94 | 4.73 | 85 | 3.84 |
| E – Amino acid transport and metabolism | 132 | 6.25 | 127 | 6.06 | 126 | 6.34 | 111 | 5.02 |
| F – Nucleotide transport and metabolism | 103 | 4.88 | 94 | 4.49 | 100 | 5.04 | 101 | 4.56 |
| H – Coenzyme transport and metabolism | 92 | 4.36 | 85 | 4.06 | 87 | 4.38 | 81 | 3.66 |
| I – Lipid transport and metabolism | 39 | 1.85 | 38 | 1.81 | 37 | 1.86 | 35 | 1.58 |
| P – Inorganic ion transport and metabolism | 98 | 4.64 | 96 | 4.58 | 92 | 4.63 | 88 | 3.98 |
| Q – Secondary metabolites biosynthesis, transport and catabolism | 22 | 1.04 | 22 | 1.05 | 22 | 1.11 | 18 | 0.81 |
| R – General function prediction only | 0 | 0.00 | 0 | 0.00 | 0 | 0.00 | 0 | 0.00 |
| S – Function unknown | 340 | 16.11 | 334 | 15.94 | 317 | 15.96 | 336 | 15.18 |
| **Total COG-assigned CDS** | 1572 | 74.47 | 1507 | 71.93 | 1425 | 71.75 | 1511 | 68.28 |

^1^ NCBI accession number: GCA_030418275.1  
^2^ NCBI accession number: GCA_000159475.2 (MM4-1a) 
^3^ NCBI accession number: GCF_000010005.1 
^4^ NCBI accession number: GCF_000159455.2

 Supplementary Table 2. Comparison of COG functional categories of *L. reuteri* strain genomes in the COG V category (Defense mechanisms). The COG category was taken from the eggNOG database. Analyzed *L. reuteri* strains: Lr4659, Lr6475, JCM 1112 and SD2112.

| **COG Description** | **COG** | **Lr4659^1^** | **Lr6475^2^** | **JCM 1112^3^** | **SD2112^4^** |
| --- | --- | --- | --- | --- | --- |
| Type I restriction-modification system | COG0286 | 2 | 2 | 2 | 1 |
| Type II restriction enzyme, methylase subunits | COG1002 | 0 | 0 | 0 | 1 |
| ABC transporter, ATP-binding protein | COG1132 | 0 | 0 | 0 | 1 |
| ABC transporter, ATP-binding protein | COG1136 | 2 | 2 | 2 | 2 |
| domain protein | COG1511 | 1 | 1 | 1 | 1 |
| Beta-lactamase | COG1680 | 2 | 2 | 2 | 2 |
| ABC transporter transmembrane region | COG2274 | 1 | 2 | 1 | 1 |
| Beta-lactamase enzyme family | COG2367 | 1 | 1 | 1 | 1 |
| EcoEI R protein C-terminal | COG4096 | 1 | 1 | 1 | 0 |
| VanZ like family | COG4767 | 1 | 1 | 1 | 2 |
| Multidrug transporter MatE | COG0534 | 2 | 2 | 2 | 2 |
| ABC-2 type transporter | COG0842 | 1 | 1 | 1 | 1 |
| ABC transporter, ATP-binding protein | COG0577 | 1 | 1 | 1 | 1 |
| Subunit R is required for both nuclease and ATPase activities, but not for modification | COG0610 | 1 | 1 | 1 | 1 |
| Type I restriction | COG0732 | 2 | 2 | 3 | 2 |
| Eco57I restriction-modification methylase | COG0827 | 0 | 0 | 0 | 1 |
| **Total** |  | 18 | 19 | 19 | 20 |

^1^ NCBI accession number: GCA_030418275.1  
^2^ NCBI accession number: GCA_000159475.2 (MM4-1a) 
^3^ NCBI accession number: GCF_000010005.1 
^4^ NCBI accession number: GCF_000159455.2

Supplementary Table 3. API 50 CH result of the strain Lr4659.

| **Substrate** | **Result^*^** | **Substrate** | **Result^*^** |
| --- | --- | --- | --- |
| Glycerol | - | Salicin | - |
| Erythritol | - | D-Cellobiose | - |
| D-Arabinose | - | D-Maltose | + |
| L-Arabinose | + | D-Lactose | + |
| D-Ribose | + | D-Melibiose | + |
| D-Xylose | - | D-Saccharose | + |
| L-Xylose | - | D-Trehalose | - |
| D-Adonitol | - | Inulin | - |
| β-Methyl-D-Xylopyranoside | - | D-Melezitose | - |
| D-Galactose | + | D-Raffinose | + |
| D-Glucose | + | Amidon | - |
| D-Fructose | - | Glycogen | - |
| D-Mannose | - | Xylitol | - |
| L-Sorbose | - | Gentiobiose | - |
| L-Rhamnose | - | D-Turanose | - |
| Dulcitol | - | D-Lyxose | - |
| Inositol | - | D-Tagatose | - |
| D-Mannitol | - | D-Fucose | - |
| D-Sorbitol | - | L-Fucose | - |
| α-Methyl-D-Mannopyranoside | - | D-Arabitol | - |
| α-Methyl-D-Glucopyranoside | - | L-Arabitol | - |
| N-AcetylGlucosamine | - | Potassium gluconate | + |
| Amygdalin | - | Potassium 2-Ketogluconate | - |
| Arbutin | - | Potassium 5-Ketogluconate | - |
| Aesculin | - |  |  |

^*^ +: positive reaction, -: negative reaction
