# Supplementary figures and images for: Genomic, phenotypic, and clinical safety of Limosilactobacillus reuteri ATCC PTA 4659
Source: J Ind Microbiol Biotechnol. 2023 Nov 16;50(1):kuad041. doi: 10.1093/jimb/kuad041 (PMC10689046; doi:10.1093/jimb/kuad041)

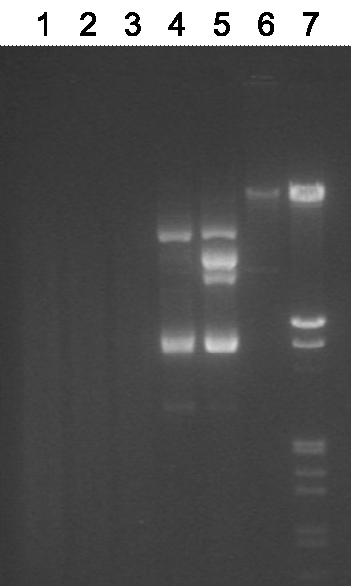

Supplement: kuad041_Supplemental_File [file kuad041_supplemental_file.zip › suppl_fig_1.tiff]

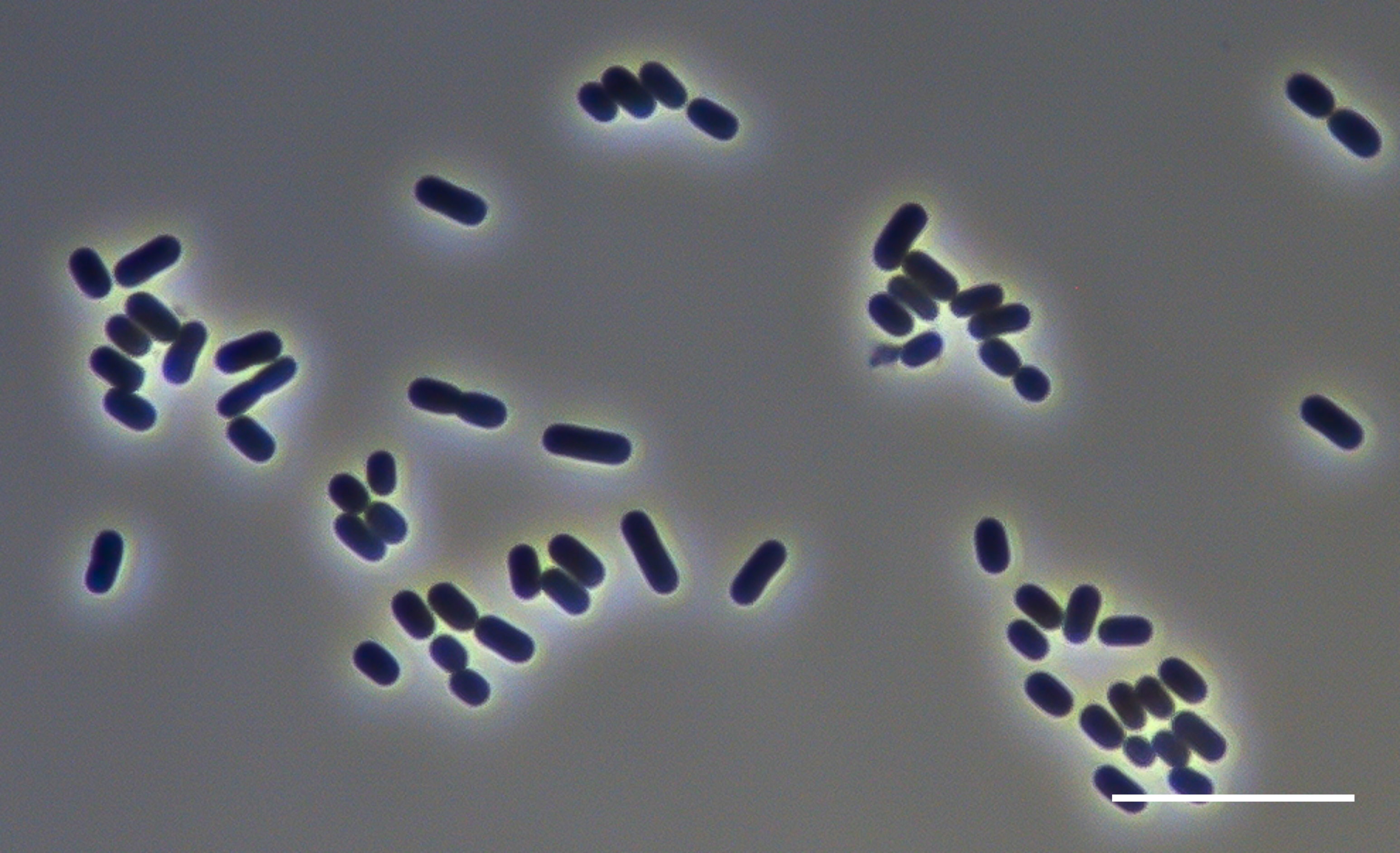

Supplement: kuad041_Supplemental_File [file kuad041_supplemental_file.zip › suppl_fig_2.tiff]
